# Dry Eye Disease: From Mechanisms to Management and Future Directions

**DOI:** 10.3390/jcm15072535

**Published:** 2026-03-26

**Authors:** Zofia Pniakowska, Natasza Kurys, Hanna Pietruszewska, Aleksandra Przybylak, Piotr Jurowski

**Affiliations:** 1 Department of Ophthalmology and Vision Rehabilitation, Medical University of Lodz, Ul. Zeromskiego 113, 90-549 Lodz, Poland; 2 Optegra Eye Clinic, Ul. Skladowa 35, 90-127 Lodz, Poland

**Keywords:** dry eye disease, tear film homeostasis, ocular surface inflammation, meibomian gland dysfunction, diagnosis, treatment

## Abstract

Dry eye disease (DED) is a complex, multifactorial, progressive disease that has consequences both for individuals and society. Symptoms reported by patients include discomfort in the eye and periodic blurred vision, while in the broader perspective, the disease is associated with economic burdens and challenges for healthcare systems. Globally, dry eye disease remains a growing problem observed in many countries. It is estimated that symptoms of dry eye syndrome occur in approximately 10 to 20 per cent of people over the age of 40. This prevalence is on the rise, which is associated with both the aging population and increased incidence among younger adults. In this group, factors such as contact lens wear and prolonged use of digital devices are considered to be contributing factors. Further epidemiological studies, conducted in different regions of the world, covering diverse populations and a wide range of age groups, with a particular focus on younger cohorts, may contribute to a more accurate understanding of the prevalence of dry eye disease. There are more and more methods of diagnosing DED. In addition to well-known procedures like the Schirmer test or tear break-up time, there are also methods that focus on the evaluation of the tear film or imaging of the ocular surface. Moreover, usage of artificial intelligence is also playing a significant role in it. However, the key issue in individual cases is introducing the most effective treatment based on combining available substances, including corticosteroids, antibiotics and supplements, which leads to a reduction in inflammation and improvement in visual comfort.

## 1. Introduction

Dry eye disease (DED) is one of the most common diseases of the ocular surface and is a growing public health problem worldwide. According to epidemiological studies, the prevalence of DED in the general population ranges from approximately 5% to over 30%, depending on the diagnostic criteria used, the age of the population studied, and environmental and cultural factors [1]. It fluctuates between 8.7 and 11.0% in Caucasian populations and 16.7 and 33.4% in Eastern Asia. Additionally, DED occurs more frequently in women. It is also believed that the risk increases with age but there are more and more cases of the disease among 18–50-year-old age groups because of commonly worn contact lenses and worldwide-used electronic devices like smartphones or computers [2]. The TFOS DEWS II Task Force (Tear Film and Ocular Surface Society Dry Eye Workshop II Task Force) proposed an updated definition of dry eye disease, describing it as a disease with a complex etiology, involving the ocular surface and leading to tear film imbalance [3,4]. This process is accompanied by symptoms reported by patients, and its development is associated with tear film instability, increased osmolarity, inflammatory response, and damage to the barrier of the ocular surface. The course of the disease also involves abnormalities in neurosensory mechanisms, which may affect the nature and severity of clinical symptoms, as stated in the report of the TFOS DEWS II Subcommittee on Definitions and Classification and developed in the report of TFOS DEWS III [3,4,5]. The aim of the manuscript is to illustrate the pathomechanism and risk factors of DED and to summarize the methods of diagnosis and treatment, including promising solutions that require further development, like smart contact lenses, emulsions, dendrimers or nanomicelles.

## 2. Materials and Methods

We searched articles in the following databases: PubMed, Scopus and Google Scholar. The chosen research papers were published between 1995 and 2026. We focused on the latest studies, especially from the last five years. The phrases typed into the search engine were: ‘DRY EYE DISEASE’, ‘DRY EYE SYNDROME’, ‘DRY EYE’, ‘OCULAR SURFACE’, ‘OCULAR SURFACE DISEASE’, ‘OCULAR SURFACE INFLAMMATION’, ‘TEAR FILM’, ‘TEAR FILM HOMEOSTASIS’, ‘MEIBOMIAN GLAND DYSFUNCTION’.

## 3. Pathomechanism

The surface of the eye is covered with a continuous layer of epithelium, including the cornea, the anterior segment of the eye and the conjunctiva, which extends to the skin–mucosal junctions of the eyelid margins. Tears provide adequate hydration of the ocular surface, forming a uniform film over its exposed areas. Tears are secreted primarily in the lacrimal glands, with the participation of conjunctival structures, including goblet cells and meibomian glands. The surface of the eye is constantly exposed to factors that promote drying, mainly related to tear evaporation [6]. It is protected from damage by homeostatic mechanisms that adjust the quantity and distribution of tears in response to stimuli reaching the surface of the eye. In dry eye disease, these mechanisms fail, resulting in a quantitative or qualitative deficiency of tears [7,8]. The consequences of these disorders are: tear film instability, impaired wetting of the eye surface, hyperosmotic stress, increased friction and prolonged mechanical irritation of the tissues. These phenomena initiate a sequence of inflammatory reactions and damage to the eye surface typical of this condition. The main lacrimal gland has a tubulo-lobular structure and is serous in nature. It is composed primarily of lobular, ductal and myoepithelial cells, with lobular cells accounting for approximately eighty per cent of its structure. In addition to the main gland, there are also additional lacrimal glands. Approximately forty Krause glands are located in the upper conjunctival vault, while six to eight are located in the lower vault. Wolfring’s glands, slightly larger than Krause’s glands, occur in numbers ranging from two to five in the upper eyelid and from one to three in the lower eyelid. Additional lacrimal glands have a tubular structure and, unlike in some animal species, do not contain acini in humans. Together, they account for about ten per cent of the total mass of the glandular tissue of the lacrimal system and have a similar innervation pattern to the main gland, suggesting a similar response to reflex stimuli.

There is a clear correlation between the incidence of DED and age. Numerous population studies indicate that the risk of developing DED increases with aging, which is associated, among other things, with a decrease in lacrimal gland function, hormonal changes and coexisting systemic diseases. The disease affects women more often than men, which is explained by the influence of sex hormones, especially estrogens and androgens, on the functioning of the tear film and meibomian glands [2]. The development and progression of dry eye disease is associated with a number of systemic medications, including antihistamines, antidepressants, anxiolytics, isotretinoin, and hormone replacement therapy [9]. There has also been an increase in the incidence of DED in younger age groups, which is associated with the intensive use of digital devices, reduced blinking frequency and prolonged exposure to artificial lighting and air-conditioned working environments [2]. Environmental factors such as air pollution, low humidity and exposure to tobacco smoke also play an important role. Dry eye disease often co-occurs with autoimmune diseases, in particular Sjögren’s syndrome, rheumatoid arthritis and systemic lupus erythematosus, as well as dermatological diseases such as rosacea. DED can also be a complication of pharmacotherapy, especially when using antihistamines, antidepressants, beta-blockers or oral retinoids [9]. The current understanding of the pathomechanism of dry eye disease is based on the definition proposed by the Tear Film & Ocular Surface Society (TFOS DEWS III), according to which DED is a multifactorial disease of the ocular surface characterized by the loss of tear film homeostasis, accompanied by ocular symptoms, tear film instability, hyperosmolarity, inflammation and damage to the ocular surface [5]. The central element in the pathogenesis of DED is the disruption of the stability of the tear film, which under physiological conditions performs protective, nutritional and optical functions.

The tear film consists of a lipid layer, an aqueous layer and a mucin layer, and dysfunction of any of these layers can lead to accelerated tear evaporation or insufficient tear production. A lipid layer is responsible for lubrication of the ocular surface, reduction in friction during blinking and prevention of tears’ evaporation. It consists of different lipid and protein components, like free fatty acids, waxes, triglycerides or cholesteryl esters, which are secreted by meibomian glands [10]. An aqueous layer is maintained due to tear production by lacrimal glands in the upper corner of the eyeball. It is mainly composed of water, electrolytes, glucose and antibacterial proteins, like lysozyme or lactoferrin. Its role is the protection of the ocular surface and supplying nutrients to the cornea [11]. The innermost layer—mucin layer—is constituted by glycoproteins produced by conjunctival goblet cells that provide tear film stability and appropriate distributing of water [7]. On this basis, two main mechanisms of the disease are distinguished: aqueous-deficient dry eye and evaporative dry eye, although in clinical practice, they often coexist in the same patient. One of the pathophysiological processes is hyperosmolarity of the tear film, leading to the activation of an inflammatory cascade on the surface of the eye. Elevated electrolyte concentrations induce osmotic stress in corneal and conjunctival epithelial cells, resulting in the release of pro-inflammatory cytokines, matrix metalloproteinases (MMPs) and pro-apoptotic factors. This process leads to damage to goblet cells, reduced mucin production and further destabilization of the tear film. Chronic inflammation, which is a self-perpetuating vicious circle, is also part of the pathomechanism of DED. Damage to the surface of the eye promotes the activation of the immune response, including T lymphocytes, which exacerbate the inflammatory process and lead to further deterioration in the function of the lacrimal and meibomian glands. The most significant reason for evaporative DED is meibomian gland dysfunction (MGD) connected with obstruction of the terminal duct and impaired meibum secretion, which can be caused by endogenous and environmental factors like age, gender, level of hormones and taken medicaments. Hyperkeratinization of the ductal epithelium and increased density of the secretion cause the blockade of the glands’ orifices, which leads to insufficient composition of the lipid layer, increased tears’ evaporation and inflammation of the ocular surface [12]. The pathogenesis of dry eye disease also emphasizes the role of neurosensory disorders, consisting of the abnormal transmission of sensory stimuli from the surface of the eye [5]. The correct structure of corneal nerves can be disrupted by factors like injuries, medical procedures, the aging process or different diseases, like diabetes [13]. These damages may lead to modified sensation, ocular pain and the development of DED. Altered sensation can also be a result of functional disorders of corneal nerves caused by incorrect activity of growth factors and overexpression of sodium channels, which disrupt blinking and appropriate tear production [5]. Moreover, dysfunction of the central or peripheral nervous system does not remain without influence on the effective transmission of sensory impressions from the ocular surface to the cortex. This may result in severe subjective symptoms with minor clinical changes or, conversely, significant damage to the surface of the eye with few patient complaints.

Clinical and laboratory studies conducted over the last few decades indicate that dry eye disease is a chronic inflammatory disease. Its development can be triggered by various environmental or endogenous factors that contribute to the formation of an unstable and hyperosmotic tear film. The modified composition of tears, sometimes coexisting with systemic conditions, leads to the activation of an inflammatory mechanism, resulting in damage to the surface epithelium of the eye and stimulation of nerve structures. Sudden drying of the surface of the eye activates stress response pathways in epithelial cells and resident immune system cells. This process initiates the synthesis of inflammatory mediators that are part of the innate response, which promotes increased expression of matrix metalloproteases, the influx of inflammatory cells and the maturation of dendritic cells [4]. The mediators produced, in combination with exposure to autoantigens, can lead to the development of a T-cell-dependent adaptive response.

Damage to the corneal barrier occurs as a result of the degradation of tight junctions between epithelial cells, which occurs with the participation of proteases [4]. This results in accelerated cell death, exfoliation, the formation of an uneven and insufficiently moisturized corneal surface, and exposure and hypersensitivity of epithelial nociceptors. At the same time, dysfunction and loss of conjunctival goblet cells are observed, which are promoted by the action of interferon gamma, a type 1 T-cell cytokine. The changes occurring in the epithelium further destabilize the tear film and intensify the inflammatory process, leading to the perpetuation of a self-sustaining mechanism. The treatment of dry eye disease includes cyclosporine and lifitegrast, preparations approved for use by the US Food and Drug Administration, which limit T-cell activation and cytokine production. Despite advances in treatment, the effectiveness of these methods remains limited in some patients, especially in terms of reducing symptoms and corneal epithelial changes [4]. Preclinical studies indicate the existence of additional therapeutic targets, potential biomarkers and opportunities to enhance natural immunoregulatory mechanisms. Further development of knowledge in this area is expected to contribute to the improvement in diagnostics and treatment methods for DED [4].

The relationship between the use of cosmetics around the eyes and the development of dry eye disease has been described in numerous studies in which the presence of tear film contamination was observed during routine assessment of the eye surface. Daily habits such as rubbing the eyes, incorrect application techniques, spontaneous migration of cosmetics into the conjunctival sac and the accidental application of products directly to the eyelid margin contribute to DED [1]. In addition to benzalkonium chloride, other substances commonly found in make-up products have an adverse effect on the continuity and functioning of the eye surface. This group includes chlorphenesin, formaldehyde-releasing compounds, parabens and phenoxyethanol, which are commonly used in skin care creams, and adhesives used to apply false eyelashes. Eyelash extension treatments can also lead to inflammatory reactions of the eye surface and inflammation of the cornea and conjunctiva, both as a result of the adhesive getting into the eye and during its removal. Retinoids, which are derivatives of vitamin A widely used in anti-aging preparations, have also been shown to have a negative effect on the functioning of the meibomian glands. The penetration of cosmetics into the tear film through the eyelid margin can result in numerous ophthalmic consequences, including inflammation of the posterior eyelid margin, reduced tear film stability, conjunctival pigmentation changes, irritation of the eye surface and keratitis [14].

The mechanisms underlying these disorders include the presence of impurities in the lipid layer of the tear film, dysfunction of the meibomian glands and their mechanical closure, which promotes increased tear evaporation and deterioration in the tear film quality. Botulinum toxin is widely used in aesthetic medicine, and its injections into the medial part of the upper and lower eyelids have also been used in the treatment of dry eye syndrome symptoms [15]. The mechanism of action of this procedure involves limiting the activity of the orbicularis oculi muscle in the medial part, which promotes longer retention of tears on the surface of the eye. At the same time, cases of deterioration in tear film stability after the use of botulinum toxin have been reported, with higher doses associated with more severe effects. The long-term effect of repeated botulinum toxin injections on the condition of the eye surface remains unclear [16].

## 4. Symptoms of Dry Eye Disease

Dry eye disease is a chronic disorder of the ocular surface in which subjective symptoms play a central role in both the diagnostic process and the assessment of treatment outcomes. A characteristic feature of DED is its marked clinical heterogeneity and the frequent discordance between patient-reported symptoms and objective clinical signs, which constitute a significant challenge in both clinical practice and research. The symptoms of DED arise primarily from tear film instability, increased tear osmolarity, and neurosensory abnormalities of the ocular surface, and they may fluctuate considerably over time as well as in response to environmental and behavioral factors [17,18].

### 4.1. Sensory Symptoms and Ocular Surface Discomfort

The most commonly reported symptoms include sensations of dryness, burning, stinging, irritation, and foreign body sensation. These complaints are directly related to tear film instability and a reduced tear film break-up time (TBUT), particularly in patients with the short TBUT-type DED phenotype, which is characterized by minimal structural changes in the ocular surface despite pronounced subjective symptoms. This phenotype has been reported most frequently in Asian populations, although its clinical relevance is increasingly recognized worldwide [17,19].

### 4.2. Visual Disturbances and Visual Function Impairment

Visual symptoms represent a significant component of the clinical presentation of DED and include fluctuating or unstable vision, transient blurred vision, and difficulty maintaining visual focus. These symptoms result from irregularities of the tear film covering the ocular surface and may lead to reduced contrast sensitivity and subjective deterioration in visual quality, even when standard measurements of visual acuity remain within normal limits. Such visual disturbances have substantial functional implications, potentially affecting activities such as driving and tasks requiring sustained visual precision [20,21].

### 4.3. Paradoxical Tearing and Masking Symptoms

In some patients with DED, excessive tearing is observed as a reflex response to ocular surface irritation, which may obscure the underlying tear film dysfunction and delay accurate diagnosis. This phenomenon is particularly common in younger individuals and contact lens wearers, in whom symptoms of dry eye may coexist with apparently preserved tear production on quantitative testing [22,23].

### 4.4. Symptoms Associated with Digital Screen Exposure

Increasing attention has been directed toward the role of prolonged digital screen use in the development and exacerbation of DED symptoms. Reduced blink rate and incomplete blinking during screen use contribute to increased tear evaporation, resulting in symptoms such as ocular fatigue, eyelid heaviness, burning sensations, and transient visual disturbances. These manifestations are particularly prevalent among children, adolescents, and young adults and are reflected in the rising prevalence of symptomatic DED in younger age groups [24,25].

### 4.5. Pain-Related Symptoms and Neurosensory Abnormalities

In a subset of patients, pain-related symptoms dominate the clinical picture, including photophobia, burning ocular pain, hypersensitivity to mechanical or chemical stimuli, and persistent ocular discomfort. These symptoms may occur independently of the severity of classical clinical signs and are often associated with abnormalities in corneal nerve function and sensory processing, leading to a pronounced discordance between symptoms and objective findings [17,18].

### 4.6. Impact of DED Symptoms on Mental Health and Quality of Life

A growing body of evidence demonstrates a significant association between the severity of DED symptoms and reduced quality of life, as well as increased prevalence of mood disorders, anxiety, and depressive symptoms. Meta-analyses indicate that patients with DED are more likely to experience comorbid psychiatric conditions, and this relationship appears to be bidirectional, underscoring the importance of a holistic, patient-centered approach to disease management [21,26,27].

### 4.7. Demographic and Clinical Factors Influencing Symptom Severity

The severity and nature of DED symptoms vary according to sex, age, and the presence of specific risk factors. Women more frequently report greater symptom severity compared with men, which may be related to hormonal influences and sex-specific differences in pain perception. Moreover, increased symptom burden has been observed in individuals with myopia, contact lens wearers, and patients using systemic medications, further highlighting the heterogeneous nature of DED and the need for individualized diagnostic and therapeutic strategies [23,28,29,30].

The discrepancy between subjective symptoms and objective signs of dry eye disease (DED) is a common and clinically significant phenomenon. Patients may report severe discomfort despite minimal findings on clinical examinations, or conversely, they may exhibit pronounced signs of the disease with mild or absent symptoms [18].

Multicenter studies, including the DREAM study, have confirmed that the correlation between symptom severity (measured by OSDI) and clinical signs, such as tear film break-up time (TBUT), Schirmer test, or ocular surface staining, is often weak. Changes in symptoms over time do not always align with changes in clinical signs [17].

The short TBUT phenotype, characterized by minimal structural changes on the ocular surface despite pronounced subjective symptoms, requires particular attention when planning treatment and monitoring therapeutic outcomes [19].

**Figure 1:**
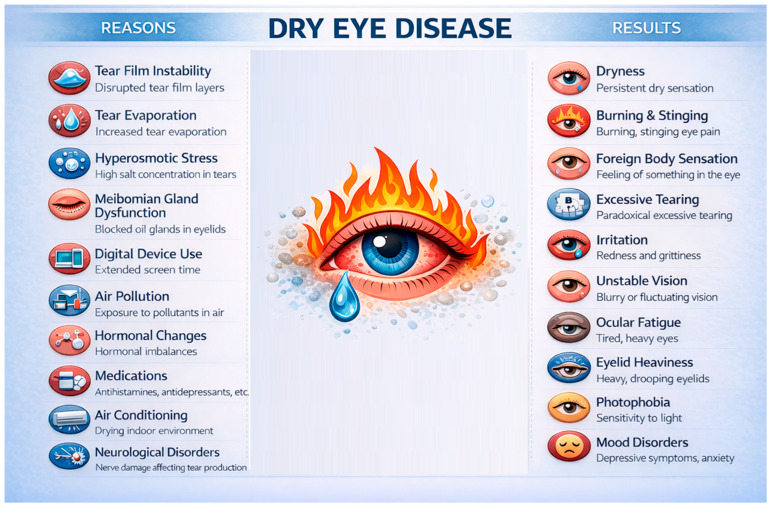
Graphical presentation of reasons and results of DED.

Additionally, in younger patients and contact lens wearers, masking symptoms such as paradoxical tearing may occur, which can falsely suggest normal tear film function [22]. The summary of reasons and results of DED is illustrated in Figure 1.

## 5. Diagnosis of Dry Eye Disease

The diagnosis of DED represents a significant clinical challenge due to the multifactorial pathogenesis of the disease, its variable course, and frequent discrepancies between subjective symptoms and objective findings. Modern diagnostic approaches rely on the integration of clinical data, functional tests, imaging techniques, and increasingly, digital tools and artificial intelligence, reflecting the dynamic evolution of knowledge in this field [31,32].

### 5.1. Evolution of the Diagnostic Approach

Historically, DED diagnostics focused primarily on the quantitative assessment of tear production and the presence of ocular surface damage. However, this approach proved insufficient, as it did not account for tear film instability, inflammatory components, or neurosensory dysfunctions. The introduction of the concept of tear film homeostasis disruption as a central feature of DED led to a paradigm shift in diagnostics, emphasizing the assessment of both subjective symptoms and multiple tear film and ocular surface parameters [31,33].

### 5.2. Importance of Symptom Assessment

Current diagnostic algorithms highlight the role of subjective symptoms as the starting point for DED diagnosis. Standardized questionnaires enable the identification of symptomatic patients and assessment of disease impact on daily functioning. Numerous studies, however, indicate that symptoms do not always correlate with clinical severity, emphasizing the need for objective methods to confirm the diagnosis and characterize the disease phenotype [32,34].

### 5.3. Classical Diagnostic Tests

The primary tests used in DED diagnosis provide information on tear film stability, aqueous tear secretion, and ocular surface damage. Their use, however, is limited by invasiveness, variability, and susceptibility to environmental and technical factors, which can result in the underestimation of early or mild disease [35,36].

#### 5.3.1. Schirmer Test

Schirmer Test I

Purpose: Assess the quantity of tears produced over a set period. It is a test that enables measuring basal and reflexive tear secretion [37].

Method: A Schirmer paper strip is placed in the lower conjunctival fornix for 5 min, and the length of the wet portion is measured. Patient is obliged to close his eyes for 5 min.

Interpretation: After 5 min, the examiner checks the extent of the moistened surface of the strip. More than 15 mm means that tear secretion is correct. Results between 10 and 15 mm indicate the onset of dry eye disease. Values between 5 and 10 mm prove a significant tear deficiency. A wet surface smaller than 5 mm is related to advanced DED.

Advantages: Simple, inexpensive, widely used.

Limitations: Invasive; may cause irritation and tearing, affecting results; low repeatability [35].

Schirmer Test II

This procedure assessing the secretion of the aquatic component of the tear film is quite similar to the Schirmer Test I but it measures only basal tear production [37]. The patient has anesthetic drops applicated to the conjunctival sac beforehand to eliminate the irritation’s influence of the result. Afterwards, the paper strip is located in the lower conjunctival fornix. Interpretation of the result is analogous to the method mentioned above.

#### 5.3.2. Tear Break-Up Time (TBUT)

Purpose: The aim of the test is the evaluation of tear film stability, quality of the lipid component of the tear film and susceptibility to the evaporation of and damages to the ocular surface.

Method: After fluorescein instillation, the cornea is observed under a slit lamp, and the time until the first break in the tear film appears is measured.

Interpretation: TBUT < 10 s indicates an unstable tear film. A result between 5 and 10 s testifies about the increased risk of DED development. TBUT < 5 s is connected to advanced impairment of moisturizing.

Advantages: Simple and widely used clinically.

Limitations: Invasive; results depend on the operator and environmental conditions [35,36].

#### 5.3.3. Ocular Surface Staining

Purpose: The method is used in detecting corneal and conjunctival epithelial damage [37].

Method: Dyes such as fluorescein, lissamine green, or rose bengal are applied.

Observation with a slit lamp assesses the degree and location of epithelial defects. The dye penetrates the structure of damaged tissue. Predominantly, yellow fluorescein is used in visualizing damaged corneal cells and lissamine green exposes necrotic conjunctival cells.

Interpretation: Pronounced staining indicates ocular surface damage; severity can be graded using scoring systems. In most cases, more than 10 tinted spots are related to noticeable damages.

Advantages: Provides visual information on surface damage.

Limitations: Partially invasive; requires clinical expertise [35].

#### 5.3.4. Lid-Parallel Conjunctival Folds Scale (LIPCOF Scale)

Purpose: This is a tool useful in assessing the presence of conjunctival folds situated parallelly to the lower lid margin [38].

Method: The number of folds is noticed by the examiner observing the ocular surface by a slit lamp microscope. The folds develop in the condition of insufficient moistness, which contributes to a weakened connection between conjunctiva and the basal layer. Numerous folds correlate with intensive scraping and the advancement of DED.

Interpretation: There are five degrees, wherefore the upper number means a higher intensity of DED.

0: Lack of folds;
1: One little fold is present;
2: There are a few little folds;
3: Numerous folds that reach to half of the lower lid;
4: Numerous folds that cover the entire length of the lower lid.

#### 5.3.5. Tear Osmolarity Measurement

Purpose: The aim is to assess the electrolyte concentration in the tear film, an indicator of hyperosmolarity characteristic of DED [37,39].

Method: A micro-sample (nanoliters) of tears is collected from the conjunctival sac and analyzed using microsensors or osmometry tests, like an I-Pen, which even performs 250 single measurements per second [39].

Interpretation: An increased electrolyte concentration is mostly connected with insufficient tear production or excessive evaporation of lacrimal film. It predominantly contributes to the inflammation and destruction of the ocular surface. A result >308 mOsm/L indicates hyperosmolarity, >316 mOsm/L correlates with advanced DED; larger inter-eye differences suggest tear film instability.

Advantages: Objective biochemical parameter; correlates with disease severity.

Limitations: Expensive; requires precise sample collection [36].

### 5.4. Non-Invasive Tear Film Assessment

Significant progress in DED diagnostics has been achieved through non-invasive tear film evaluation methods, including non-invasive tear break-up time (NIBUT), lipid layer interferometry, tear meniscus height measurement, and dynamic tear film analysis using topographic systems [40,41].

### 5.5. Ocular Surface Imaging

Modern imaging techniques, including meibography and advanced corneal imaging modalities, play an increasingly important role in the diagnosis and management of DED. Meibography allows detailed visualization of the meibomian glands, enabling clinicians to assess both the structural integrity and functional status of these glands, which are essential for maintaining a stable tear film and preventing evaporative dry eye. Advanced corneal imaging, such as high-resolution topography and optical coherence tomography, permits the precise evaluation of corneal surface irregularities, tear film distribution, and epithelial damage. These methods provide objective, reproducible data crucial for differentiating between DED subtypes, guiding therapeutic decisions, and monitoring treatment efficacy over time. Moreover, ocular surface imaging can detect subtle changes not apparent in routine clinical examinations, allowing for earlier intervention and more individualized patient care [39,41].

### 5.6. Diagnosis of DED in Systemic Diseases

In patients with systemic autoimmune disorders, particularly Sjögren’s syndrome, DED diagnosis is of critical clinical importance. Ocular dryness may represent one of the earliest manifestations of systemic disease and is often more severe and persistent than primary DED. Comprehensive ophthalmic assessment, including tear production tests, ocular surface staining, and advanced imaging, is essential not only for confirming DED but also for evaluating severity and impact on visual function. Early detection facilitates the timely initiation of targeted therapies, collaboration with rheumatologists or immunologists, and the prevention of long-term ocular complications, such as corneal ulceration or infection. Recognizing systemic associations also helps tailor management plans and optimize overall quality of life [42].

### 5.7. Assessment of Disease Severity and Multidimensional Approach

Accurately assessing disease severity is a major challenge in DED management. Current guidelines recommend a multidimensional approach integrating patient-reported symptoms, clinical test results, tear film parameters, and the impact on visual function. This method captures the heterogeneous nature of DED, including aqueous-deficient, evaporative, and mixed forms. By combining subjective and objective measures, clinicians can stratify patients, personalize therapy, and monitor treatment response over time. Multidimensional assessment is particularly important for guiding advanced interventions, such as anti-inflammatory therapy, punctal occlusion, or gland-targeted treatments, ensuring optimal functional outcomes and patient satisfaction [33,34].

### 5.8. The Role of Artificial Intelligence in DED Diagnosis

AI represents a highly promising direction in DED diagnostics. Machine learning (ML) algorithms can automatically analyze ocular surface images, tear film parameters, and clinical data from multiple sources, providing objective and reproducible assessments. AI systems have demonstrated high accuracy in identifying DED, classifying disease severity, estimating TBUT, and predicting phenotypes. Beyond supporting real-time clinical decision-making, AI enables longitudinal disease monitoring, early identification of treatment responders, and the development of personalized therapeutic strategies. Integrating AI into clinical practice enhances diagnostic precision, reduces inter-observer variability, and optimizes patient outcomes while laying the foundation for future automated ocular health assessments [43,44,45].

In the diagnosis and management of DED, AI utilizes advanced data analysis techniques, such as ML, deep learning (DL) and convolutional neural networks (CNNs). These technologies allow the automatic processing of large datasets, including ocular surface images, tear film parameters, and clinical data, significantly enhancing the objectivity and reproducibility of patient assessments [45]. AI-assisted meibography analysis enables the detection of subtle changes in the meibomian glands, such as gland atrophy, dropout, or abnormal morphology, which might be overlooked in traditional manual evaluation [45]. CNNs allow automatic feature extraction from images, such as tear meniscus height, corneal staining, or lipid layer quality, enabling the rapid and precise classification of DED severity without manual intervention [44]. AI can identify both aqueous-deficient and evaporative DED phenotypes, providing a more detailed assessment and facilitating personalized treatment strategies. AI also plays a key role in the predictive modeling of DED progression. ML and DL algorithms analyze clinical data, including patient-reported symptoms, Schirmer test results, TBUT, and inflammatory biomarkers, to predict disease progression and the likely effectiveness of different therapies [43]. Clinical studies have shown that AI improved diagnostic accuracy by 15–20% compared to traditional methods [45].

AI models also demonstrated high reproducibility in DED severity classification, with image processing times reduced to less than one second per meibography image, compared to several minutes for manual evaluation [44]. Clinical case results highlight the practical benefits of AI in DED management. In one analysis, patients evaluated with the AI-assisted assessment of meibomian glands and tear film stability achieved better therapy matching, resulting in an average TBUT improvement of 2.1 s and a 20% reduction in subjective dry eye symptoms (OSDI score) over 3 months [46]. In another case, patients with advanced meibomian gland dysfunction assessed by AI received a personalized combination of pharmacologic therapy and thermal treatments, leading to symptom relief and improved tear film quality compared to a control group receiving standard therapy [46].

AI also enables the monitoring of patients between clinic visits using digital systems, mobile applications, or high-resolution image analysis, allowing the early detection of symptom worsening and timely adjustments to therapy [46]. The integration of multimodal data—including meibography, tear film analysis, clinical test results, and patient demographics—allows the creation of more precise predictive models and treatment recommendations, increasing therapeutic efficacy and minimizing errors [40]. Despite its advantages, AI implementation in clinical practice presents challenges, such as the lack of standardized diagnostic criteria, variability in data quality, limited diversity in patient populations, and ethical and privacy concerns [45]. Moreover, AI cannot fully replace clinicians in providing empathy, patient communication, or psychological support, which remain crucial in managing chronic ocular diseases like DED. In conclusion, AI represents one of the most promising tools for the diagnosis and management of DED. These systems offer high diagnostic precision, the ability to monitor disease in real time, support in personalizing therapy, and prediction of disease course. Clinical results demonstrate tangible improvements in both objective tear film parameters and subjective patient-reported outcomes, ultimately enhancing treatment results and patient quality of life (Table 1) [45].

## 6. Treatment

DED is a disease that requires long-term treatment and the use of various methods, both pharmacological and non-pharmacological, due to the diversity and multitude of possible causes. It should be introduced gradually, starting with traditional therapies and progressing to more advanced ones depending on the patient’s state. The main objective is to reduce clinical signs, stabilize the ocular surface, and prevent it from current damage, resulting in improving vision and patients’ quality of life [47].

Although the application of artificial intelligence and modern imaging technologies in the diagnosis of DED is highly promising, the literature highlights significant limitations related to their implementation in clinical practice. One of the main challenges is the high cost of technology and digital infrastructure, which may limit accessibility, particularly in centers with limited financial resources and in low-income countries [48].

Another barrier is limited accessibility and the requirement for specialized equipment and technical expertise, which complicates the integration of these solutions into routine clinical practice outside highly specialized centers [49].

An additional concern is the insufficient validation of AI models across diverse patient populations, which raises the risk of diagnostic bias due to unrepresentative training datasets and limited population diversity [49].

Furthermore, the lack of standardized diagnostic protocols and the need for further large-scale clinical validation studies remain important challenges before these technologies can be widely adopted in the diagnosis of ocular surface diseases, including DED [50].

### 6.1. Environmental and Behavioral Changes

There is a great range of behavioral and environmental changes that can help patients with DED to improve their daily living. Modifying the surroundings, such as maintaining adequate indoor humidity or reducing exposure to drying conditions, might help reduce the intensification of the symptoms. Common triggers include using air conditioning or fans, opening windows, or prolonged time in front of the computer [6]. Appropriate actions should be implemented in the patient’s life to control the disease, such as regular eyelid hygiene to reduce allergens, irritants and microbial accumulation, as well as taking scheduled breaks from the screen to relax, blink more naturally and to distribute the tear film properly. In addition, supporting overall health through proper hydration, reducing stress, being physically active, eating balanced meals, and getting enough sleep can reduce the severity of symptoms. All the above lifestyle and environmental recommendations are typically introduced as a first line of treatment; however, they require consistent adherence to them to achieve long-term therapeutic benefits [51].

### 6.2. Medical Treatment

First-line, second-line and adjunctive pharmacological methods of treatment in DED are presented in Table 2 and further explained in the text below.

#### 6.2.1. Substitution for Tears

##### Artificial Tears

Being the first line of therapy, artificial tears are local ophthalmic preparations intended to relieve dry eyes by adding moisture on the eye surface, which helps with discomfort and supports tear film function. Artificial tears help restore balance in the tear film, and improve surface hydration, stability, and spreading. Moreover, they lower ocular surface osmolarity by diluting pro-inflammatory mediators and therefore reduce the risk of inflammation [52].

These products are categorized into two main groups: demulcents, which are made of water-soluble polymers, and emollients, which are lipid-based. The first ones are supposed to be applied into the eye surface and the second ones as oil-in-water emulsions in eye drops or on eyelids [53].

While the Food and Drug Administration (FDA) has approved specific active ingredients for artificial tears, other ones are often included in these formulations and have been shown to have a therapeutic benefit but are not officially classified as active agents due to the high cost and extensive clinical testing required for regulatory approval. As a result, they are often marked as inactive ingredients. These include osmoprotectants (such as erythritol), which reduce hyperosmotic stress on epithelial cells; humectants like sodium hyaluronate that enhance water retention on the surface; viscosity-enhancing polymers such as hydroxypropyl guar, which prolong tear residence time; and lipid components (such as mineral oils), which are designed to imitate the lipid layer [54].

##### Autologous Serum

Tear drops can also be prepared from the patient’s own blood, which is called autologous serum. These preparations contain many bioactive substances, including enzymes, immunoglobulins, and proteins such as albumin and lactoferrin. These solutions present some practical limitations, including the risk of microbial contamination, gradual degradation of active agents over time, and the requirement for repeated blood sampling. Nevertheless, clinical evidence suggests that these biologic tear substitutes can significantly help patients with severe or treatment-resistant dry eye disease [40].

#### 6.2.2. Corticosteroids

In patients with moderate to severe DED, short courses of topical corticosteroids, such as hydrocortisone, can provide meaningful relief of symptoms and have been shown to reduce ocular surface inflammation effectively. However, prolonged use is associated with well-recognized adverse effects, including increased intraocular pressure, infections and cataract formation, which means that they should be used for a short period of time, even though hydrocortisone is generally considered to be safer for severe cases [55]. Of note, lotepredrol etabonate 0.25% (Eysuvis) is approved by the FDA for short-term DED treatment (up to 2 weeks).

#### 6.2.3. Cyclosporine A

Cyclosporine A (CsA) is a peptide component formed from funguses that causes both immunosuppressive and anti-inflammatory effects by inhibiting T-lymphocyte activation and reducing the release of pro-inflammatory cytokines [56]. Clinical studies have revealed that topical CsA therapy leads to improvements in the results of the Schirmer test, fluorescein staining of the cornea, and density in conjunctival goblet cells. As a result, approximately half of treated patients experience meaningful symptom relief. However, treatment is often accompanied by local adverse effects, such as irritation or burning sensations. The short-term application of topical corticosteroids has been shown to help reduce CsA-related discomfort and help tolerate treatment [52]. FDA-approved medications with cyclosporine include Restasis (cyclosporine 0.05% emulsion), Cequa (nanomicellar cyclosporine formulation) and Vevye (water-free formulation). Although cyclosporine A can reduce inflammation on the ocular surface, the clinical response is not always predictable, as only about half of patients achieve significant improvement. Furthermore, patient compliance is average due to the delayed onset of action (2–3 months), local irritation and cost of the treatment [57].

#### 6.2.4. Antibiotics (Tetracycline and Macrolides)

Tetracycline antibiotics, such as doxycycline, and macrolides, such as azithromycin, have antimicrobial effects and can also reduce inflammation. At small intranasal doses, these agents improve the function of meibomian glands and corneal surface disorders. While generally well tolerated, higher quantities are associated with adverse effects, involving skin rash, swelling and abdominal discomfort, nausea, and vomiting [55]. During clinical trials, managing DED with long-term therapy led to improvement in corneal surface integrity, tear secretion, and overall tear film stability [58].

A macrolide mechanism has shown a reduction in the leukocyte amount on the ocular surface and lowered the expression of many pro-inflammatory substances in the skin and tear film, such as tumor necrosis factor-α, interleukins (IL-1β, IL-6, IL-8, IL-10) and nuclear factor-kB [59]. Apart from its immunologic effects, azithromycin has a direct impact on the meibomian glands by increasing lipid metabolism within epithelial cells. It helps synthase and store cholesterol, neutral lipids, and lysosomal components, which leads to healthier meibum production and release [60].

Even though oral doxycycline and topical azithromycin are used off-label for DED with MGD, these drugs, however, are not FDA-approved specifically for DED.

#### 6.2.5. Lifitegrast (Xiidra)

Lifitegrast is a small-molecule integrin inhibitor, which blocks lymphocyte function-associated antigen-1 (LFA-1). It is a molecule expressed on lymphocytes that prevents their interaction with eye surface tissues and thus suppresses immune-mediated inflammation, which is one of the causes of dry eye symptoms [61]. Evidence from many randomized controlled trials, such as one involving about 600 patients from the United States and Canada [62], shows that lifitegrast improves not only clinical signs and symptoms, but also the overall quality of life of the patient who is dealing with DED, while maintaining the safety profile [61]. However, some studies report stronger improvements in symptoms than in so-called “objective” tests. Moreover, there is still a need for long-term performance data [63].

#### 6.2.6. Omega-3 and Omega-6 Supplements

Fatty acid supplements are obtained through diet and attached to the cellular membranes throughout the body [54]. Omega-6-derived arachidonic acid serves as a precursor for pro-inflammatory components causing cellular activation, while omega-3 consumption reduces this inflammatory response by displacing omega-6 fatty acids, being its natural competitive inhibitor [64]. Clinical evidence has demonstrated a correlation between tear film instability and a higher omega-6-to-omega-3 ratio, with elevated ratios associated with deterioration in the dry eye symptoms, while supplementation with omega-3 fatty acids has shown a reduction in these symptoms [65]. As a result, topical omega-3 formulations administered as eye drops have recently emerged as a promising approach to reduce eye surface inflammation in dry eye disease, although their clinical efficacy still needs to be proven. However, larger RCTs observed conflicting results. In a DREAM study (Dry Eye Assessment and Management trial), the difference between omega-3 supplementation and placebo showed no difference in improvement in symptoms or clinical signs. Currently, omega-3 supplementation can be recommended as an adjunctive strategy, particularly for patients with MGD, but not as an effective disease-modifying therapy (Table 3) [66].

## 7. Interventional Treatment Methods

Examples of emerging and advanced therapies in DED are presented in Table 4 and further described in the text below.

### 7.1. Punctal Plugs

Punctal plugs are small medical implants used in the treatment of dry eye syndrome and other ocular surface disorders, such as punctal stenosis, or to aid in local drug delivery. Their main function is to block the tear ducts or canaliculi, which helps retain natural tears on the surface of the eye, improving hydration, tear film stability, and patient comfort. Plugs can be temporary or permanent and may be made from different materials, including silicone, collagen, hydrogel, polydioxanone, or acrylic. Temporary plugs allow clinicians to assess the effectiveness of occlusion and patient tolerance before using long-term or permanent plugs. Permanent implants are used in cases requiring prolonged tear retention. Although they are considered relatively safe, punctal plugs can be associated with complications such as extrusion, migration within the canaliculi, or the formation of pyogenic granulomas. Despite this, they remain an important non-pharmacological treatment option for dry eye syndrome and a useful tool for enhancing the effectiveness of topical ocular medications [70].

### 7.2. Amniotic Membrane Grafts/Dressings

Amniotic membrane (AM) grafts and dressings represent an effective treatment method for moderate to severe DED, especially in cases resistant to conventional pharmacological therapy. AM possesses unique biological properties, including anti-inflammatory and anti-scarring effects, as well as the ability to support corneal epithelial regeneration. The use of cryopreserved AM, both in sutured and sutureless forms, provides protection to the ocular surface, promotes the healing of damaged epithelium, and improves tear film stability. Clinical studies have shown that AM reduces the symptoms of dry eye disease, decreases fluorescein-stained epithelial defects, improves corneal nerve density and sensitivity, and enhances overall ocular surface quality. Sutureless systems, such as PROKERA, allow the easy application and replacement of AM without surgical intervention, increasing patient comfort and minimizing the risk of complications. These findings indicate that AM is a promising and safe therapeutic option for DED, particularly in cases where conventional treatments fail to achieve the desired effect, and may also support corneal nerve regeneration and epithelial restoration [71].

### 7.3. Tarsorrhaphy

It is a surgical procedure that involves the partial or complete suturing of palpebral fissure. An ophthalmologist connects margins of the upper and lower eyelid temporarily or permanently to facilitate wound healing and to protect the eyeball, especially cornea, from desiccation or injuries, for example, in cases of facial nerve paralysis when the patient is not able to close his eye. This technique is used in DED treatment when other methods prove to be ineffective [72].

### 7.4. IPL

Intense pulsed light (IPL) is an off-label treatment used mostly for DED associated with meibomian gland dysfunction (MGD). This method involves exposing skin around the eyes changed abnormally by pigmentation or telangiectasias to light, usually with a wavelength of around 500 nm. Telangiectasias can absorb light and generate heat, which leads to the coagulation of abnormal blood vessels and reduction in inflammation. IPL can also decrease bacterial overgrowth and melt inspissated meibum, improving the secretion of the meibomian gland. A standard procedure involves multiple treatment sessions 2–4 weeks apart, followed by manual expression of meibomian glands. This method improves lipid film quality and reduces tear film breakdown. Side effects are usually minor, including redness and swelling. There are a few limitations, such as cost, repeating the therapy every six months or every year and limiting the use of therapy only to patients with light skin pigmentation [73]. However, most of the studies are small, with different treatment protocols, and they lack long-term follow-up. It is difficult to compare the effects between studies, as there are no standardized parameters (wavelength, number of sessions or intervals). Moreover, there are other limitations, such as cost and limited application for patients with a darker skin type (Table 5) [74].

## 8. Future Directions

### 8.1. Smart Contact Lenses (SCLs)

One of the innovative ideas for the treatment of DED is adding into contact lenses sensors that allow the constant and non-invasive monitoring of ocular biomarkers, also during sleep [75]. While most current designs are limited to detecting a single tear component, including glucose, electrolytes or metabolic byproducts, measurement of many biomarkers would significantly increase their clinical value [76].

#### 8.1.1. Medical Value

Currently, research is being conducted mainly on the use of SCLs in measuring IOP (intraocular pressure), glucose levels, and drug release. Recently, studies have been conducted regarding the use of SCLs in the treatment of other diseases, such as DED.

Enzyme-based detection, which uses glucose oxidase, and non-enzymatic detection, which monitors the glucose level based on physical or optical changes in the lens, are the two main methods used to detect glucose in SCLs. Although initial studies have shown that non-invasive tear fluid glucose testing is possible, it was not put into clinical practice due to issues with measurement accuracy [77]. More recent studies have concentrated on enhancing real-time monitoring, sensitivity and stability. For example, Park et al. (2024) demonstrated a correlation between tear fluid glucose levels and blood glucose levels and developed SCLs that may wirelessly send data about glucose levels to a smartphone [78]. The topic of another study by Kim et al. (2022) was enzyme instability [79]. Gold and platinum nanocatalysts were added to nanoporous hydrogels to improve sensitivity and reduce measurement variability during continuous monitoring.

SCLs are a promising method for ongoing, non-invasive monitoring of IOP. As they translate the corneal deformation into measurable changes, capacitive sensors are the most widely employed of the various techniques. A recent study by Yang et al. (2022) showed that increased sensitivity can be achieved by using microstructuring and novel circuits that allow wireless monitoring in real time and, in certain situations, the simultaneous administration of medications to the eye [80]. An alternative method is strain-based sensors, which can identify mechanical distortion in the lens material and translate it into electrical signals. These sensors are made of widely used flexible, conductive materials such as metal nanowires, graphene or polymers, which improve transparency, biocompatibility, and quick reaction time [81,82,83,84].

Moreover, SCLs are used as both therapeutic and diagnostic tools, enabling controlled drug release while simultaneously tracking physiological markers to evaluate treatment response in real time. This dual functionality allows for the dynamic adaptation of therapy to the patient’s condition. Recently, a new SCL was introduced capable of releasing levofloxacin and diclofenac in the event of an elevated concentration of reactive oxygen species (ROS) levels, which occurs as a response to inflammation in the ocular tissue. Such a possibility is particularly valuable for conditions like DED and post-surgical recovery, in which inflammation may cover the entire surface of the eye [85].

#### 8.1.2. Mechanism of Power Supply

One of the strategies is to apply an inductive wireless power transfer, which uses magnetic induction between two coils, one inserted into the SCLs and the other placed externally [86] or an SCL with an adhesive antenna on the skin around the eye [87]. The external unit, powered by the portable battery, which is worn by the user, generates a magnetic field that activates the lens electronics, can power the SCL by induction and also receives data from the sensor via wireless communication, such as Bluetooth [88]. This strategy reduces the thickness and stiffness of the lens and eliminates the need for a built-in battery; however, despite the requirement of the external unit, it exposes the patient to long-term electromagnetic fields.

A different strategy is to use radio-frequency identification (RFID) and near-field communication (NFC) systems to supply power and enable data transmission by electromagnetic waves. The antenna built in the lens captures this energy, converts it into electrical power, and transmits data back to the reader, often placed in the patient’s glasses [89]. The limitations of this method contain limited power availability for continuous measurements and its loss due to inefficient antenna design or placing; also, there is a risk of overheating the eye if the power is not carefully controlled [90]. Despite these limitations, this strategy is suitable for patients requiring long-term monitoring, because it is possible to integrate a rechargeable semiconductor supercapacitor that allows continuous charging of electronic components [91].

Another approach involves placing the ultrathin, elastic solid-state batteries on the contact lens surface. These batteries are supposed to supply an uninterrupted and predictable energy source, regardless of external emitters and positioning conditions. This strategy provides better energy efficiency, due to the elimination of the need for external devices or a wireless connection; however, the presence of batteries makes the lens thicker and the need for physical integration may reduce the patient’s comfort [92]. To solve the main concern, which is toxic leakage, especially with lithium-based systems, alternative battery chemistries have been proposed that contain sodium–potassium that use tear fluid as an electrocyte, which significantly reduces the risk of harmful leakage [93].

One of the problems with existing systems is their dependence on external power sources; therefore, the ability of the lenses to charge themselves via the solar battery is a promising future solution [94]. These solar cells are designed to power biological sensors used to monitor the level of glucose and calcium within the tear fluid and convert light into electrical energy even indoors, producing a voltage of approximately 0.6 V [95].

Another new energy source is the triboelectric nanogenerator, which converts mechanical motion into electricity. The nanogenerators used in the SCL could potentially capture energy generated by natural eye movements, such as blinking. However, the current challenge is to shrink these devices to the size required to fit inside the lens [96].

Hybrid power systems have also been proposed. The proposed mechanism is supposed to combine photovoltaic components with metal–air energy activated by blinking. When the eyelid passes over the tear collection device, electrolytes in the tear fluid trigger the oxidation process, releasing electrons, which are converted into electrical energy [97].

#### 8.1.3. Limitations of SCLs

In addition to the drawbacks of any SCL system, the electronics found within the lenses remain a challenge, because they are placed on the opaque materials, most of which are made of synthetic materials with limited oxygen permeability. Unfortunately, using soft hydrogel lenses can quickly damage the device under mechanical stress, such as repeated blinking [75].

Moreover, despite their potential, tear biomarker concentrations are not enough due to the ongoing miniaturization of electronic components, because reducing the size of the device can affect the sensitivity of the sensor; biosensor development remains costly and individual variety in tear composition between patients complicates standardization. Additional challenges include deterioration in the comfort of wearing lenses due to their thickening [98].

### 8.2. New Drug Delivery Systems

Another novel therapy is new drug delivery systems (DDSs) including liposomes, dendrimers, nanomicelles, nanowafers or emulsions of different particle sizes. The main goal of these systems is to increase the bioavailability of the drug, minimize side effects, and improve patient compliance. Each DDS differs in its release profile, tissue compatibility, and interaction with the ocular surface. Nanoparticles are often made from lipid-based materials including fatty acids or triglycerides connected with polymeric carriers, while liposomes increase drug penetration by improving adherence to the ocular surface and extension of the residence time [99].

#### 8.2.1. Emulsions

Microemulsions and nanoemulsions are currently being investigated as new ocular drug delivery systems. Their ability is to improve drug diffusion and residence time on the ocular surface due to low surface tension. Microemulsions are thermodynamically stable, single-phase systems composed of water, oil, and amphiphiles. Some studies have found that microemulsions, including ones based on cyclosporine A, have potential in the treatment of DED. In studies conducted on animal models, they were more effective than conventional eye drops [100]. Nanoemulsions are biphasic systems containing nanoparticle-based oil droplets that provide a high surface-to-volume ratio. Oil-in-water nanoemulsions are frequently used to transport lipophilic drugs, such as CsA. A well-known example approved for the treatment of DED is Restasis^®^ [101]. Cationic nanoemulsions enhance drug adhesion to the negatively charged ocular surface and shorten its residence time in the cornea. However, despite promising solutions, problems related to stability and droplet aggregation still limit the use of emulsions in clinical practice; therefore, further research is required [101].

#### 8.2.2. Liposomes

Liposomes are phospholipid bilayer vesicles with the ability to adhere to the ocular surface. Therefore, they increase drug delivery, especially when modified with mucoadhesive polymers or positive surface changes [102]. Eye drops based on lipids with liposomes are currently used in mild to moderate DED treatment. Such treatment improves the stability of the tear film and reduces tear osmolarity more effectively than conventional therapy, such as artificial tears [103]. Additionally, liposomal forms of drugs such as sirolimus are being considered for the treatment of DED [104].

#### 8.2.3. Dendrimers

Dendrimers are branched, three-dimensional polymers that can keep drugs within their internal cavities and participate in surface modification with biocompatible functional groups. Their water solubility and tunable surface chemistry make them promising carriers for the treatment of DED. Polyamidoamine-based (PAMAM-based) dendrimers have demonstrated strong affinity for matrix metalloproteinase-9 and showed therapeutic efficacy in animal models with DED, while dendrimer–dexamethasone conjugates reduced inflammatory cell infiltration in rabbit studies [105].

#### 8.2.4. Nanomicelles

Nanomicelles are nanoscale colloidal carriers, which are made of amphiphilic polymers or surfactants in water environment. In the treatment of DED, the use of polymeric and surfactant nanomicelles is being considered as a method for delivering poorly water-soluble drugs such as CsA. These nanomicelles stay on the corneal surface longer, can penetrate ocular tissues better and have good biocompatibility in preclinical models [106]. Micellar formulations with CsA can achieve higher drug concentrations in ocular tissues compared to standard emulsions, often being a better choice than used emulsions such as Restasis^®^ [107]. Interestingly, OTX-101 (Cequa^®^), a surfactant-based CsA nanomicelle formulation, received FDA approval in 2018, raising the prospects for the use of nanomicellar drug delivery systems in the treatment of DED in the future [108].

#### 8.2.5. Nanowafers

Nanowafers are transparent, polymer-made discs filled with a drug designed to constantly release medication into the tear fluid over a longer period and fit the ocular surface, even during blinking, without affecting vision [109]. Studies have shown that once-daily dexamethasone nanowafers had resembling results compared to twice-daily eye drops with dexamethasone [110]. The FDA has approved this clinical treatment as a promising way to improve treatment and patient compliance in ophthalmic care [111].

### 8.3. Controversy over SCLs and New Drug Delivery Systems

New drug delivery systems and SCLs are promising innovations. Nevertheless, most evidence is preclinical or from the early phases of clinical trials. There is still a need for long-term safety, long-term efficacy and patient compliance data. For SCLS, as mentioned before, technical problems such as sensor accuracy, power supply, oxygen permeability and comfort should be resolved [112].

### 8.4. New Interventional Strategies

Moreover, there are also some new interventional strategies for DED such as salivary gland transplantation or stem cell injections directly to the lacrimal glands [113].

Salivary gland transplantation is the surgical treatment considered to be a choice for people struggling with severe, resistant to conventional therapies of DED, or for those with Stevens–Johnson syndrome or Sjögren’s syndrome. This method is based on the autologous transplantation of a salivary gland, such as the submandibular gland, which becomes the new source of tears. After the removal of the salivary gland and its surrounding vessels and ducts, it is transplanted to the periocular area. The gland duct is then fused with the conjunctival vault, allowing saliva to flow directly on the surface of the eye. Secretion, which increases the volume of the tear film, improves the hydration of the eye surface and provides symptom relief. Vascular anastomosis supplies blood flow through the graft, allowing it to secrete properly and to have a long-term survival rate. Saliva contains protective proteins in comparison to artificial tears that maintain moisture on the eye surface. Although the results are promising, this technique is technically demanding and can result in many side effects, such as loss of the graft, venous thrombosis, sialolithiasis, and duct fistula [114].

Mesenchymal stem cell (MSC) injection into the lacrimal glands is a new strategy for treating severe DED, especially for DED related to autoimmune diseases, such as Sjögren’s syndrome. MSCs are characterized by strong immunomodulatory and anti-inflammatory effects and are obtained from many sources, such as bone marrow, adipose tissue, or the umbilical cord. Injection into the lacrimal gland delivers MSCs directly to the main site of tear production. This reduces inflammation or pathological angiogenesis in the gland and stimulates the regeneration of damaged follicular and ductal cells. MSCs also affect paracrine signaling, releasing cytokines, and growth factors that increase repair and immune regulation in the tissue. As a result, this leads to improvement in tear secretion and stability of the tear film of the ocular surface [115]. Although the early results are encouraging, clinical outcomes remain inconsistent, mainly due to challenges such as graft rejection, even after the administration of local immunosuppression [116]. MSCs are best described as immune-evasive, not completely immune-privilege, despite their immunomodulatory properties and low expression of major histocompatibility complex (MHC) class II molecules. Sanabria-de la Torre et al. [117] observed that allo-MSCs may induce immune responses after repeated administration, which can lead to graft rejection.

Another limitation is the variability in MSCs, as it depends on the tissue source, donor age, and culture conditions. This complicates standardization and is a cause for inconsistent clinical outcomes. Furthermore, a lot of data come from preclinical models or phase I/II clinical trials with small sample sizes and short follow-up periods. Therefore, there is still a need for RTCs evaluating long-term safety, optimal dosing, durability of effect, and route of administration [118].

MSC therapy is regarded as safe in early trials; however, there is a risk of ectopic tissue formation, fibrosis, pro-angiogenic effects and tumors, because of their proliferative and immunomodulatory properties [119].

## 9. Conclusions

DED is a multifactorial, chronic inflammatory disorder of the ocular surface resulting from the loss of tear film homeostasis, in which tear film instability, hyperosmolarity, inflammation, epithelial damage, and neurosensory dysfunction play interdependent roles in disease initiation and progression [4,16].

Epidemiological evidence demonstrates a strong association between DED prevalence and age, female sex, systemic diseases, pharmacotherapy, and environmental exposures, with a notable rise in disease incidence among younger populations due to intensive digital device use and adverse working conditions [4,15,24,25].

Symptoms range from ocular discomfort and visual disturbances to pain-related manifestations driven by neurosensory abnormalities, significantly impairing quality of life and mental health [21,26,27]. Therapeutic management of DED requires a stepwise, individualized approach combining environmental and behavioral modifications with pharmacological and supportive treatments [43,44,45]. Artificial tears remain the cornerstone of first-line therapy, while anti-inflammatory agents such as corticosteroids, cyclosporine A, and lifitegrast target immune-mediated mechanisms central to disease pathogenesis [32,61,63,64]. Adjunctive therapies, including antibiotics with anti-inflammatory properties, autologous serum eye drops, and dietary supplementation with omega-3 fatty acids, may further improve tear film quality and ocular surface integrity in selected patients [3,28,29,65].

Despite significant advances, current treatments do not fully address disease complexity in all patients, underscoring the need for novel therapeutic strategies and improved biomarkers [16]. Emerging technologies, including artificial intelligence-assisted diagnostics, smart contact lenses for tear biomarker monitoring, advanced drug delivery systems, and regenerative approaches such as stem cell therapy, offer promising avenues for future research and clinical innovation, although substantial technical and clinical challenges remain [1,9,14,15,16,17,18,44,45,46].

Dry eye disease represents a significant and growing public health problem with a complex pathophysiology, diverse clinical presentations, and substantial impact on quality of life. Continued integration of epidemiological insights, advanced diagnostics, personalized therapeutic strategies, and emerging technologies is essential to improve disease detection, optimize treatment outcomes, and reduce the overall burden of DED on patients and healthcare systems [4,16,33,43].

## 10. Limitations

Currently, there are a wide variety of therapeutic strategies used in the management of dry eye disease. Although many interventions have been shown to improve the patient’s condition in clinical studies, there is still a need for high-quality evidence, provided by double-masked, randomized trials with placebo, for most of them. As a result, the availability of these treatments is often restricted to healthcare systems. Moreover, in clinical practice, patients frequently need combination therapy, and a significant part of them experience limited or no symptomatic relief despite numerous interventions [120]. There are also many ocular lubricants, which are the main products for DED, causing prolonged trial-and-error strategies.

While patients often want to adhere to therapy, on average, they take only 4–5 months’ worth of dry eye medication within a year. Treatment discontinuation occurs in a small proportion of patients due to ocular discomfort, such as burning sensations, and the beginning of noticeable symptom improvement is often delayed, ranging from several weeks to up to three months [121]. These challenges may be due in part to the limited ocular penetration of lipophilic and hydrophobic substances in oil-in-water emulsions, including cyclosporine [122].

## Figures and Tables

**Table 1:** Applications of artificial intelligence (AI) methods in the diagnosis and management of dry eye disease (DED).

AI Method	Application in DED	Clinical Example/Result	Reference
ML	Analysis of clinical data, disease progression prediction, therapy outcome forecasting	Analysis of Schirmer test results, TBUT, and inflammatory biomarkers to predict DED progression and treatment efficacy	[45]
DL	Advanced analysis of large datasets, automatic detection of ocular surface changes	DL models analyzing TBUT and corneal staining images improved DED diagnostic accuracy by 15–20%	[44]
CNNs	Automatic feature extraction from meibography, tear meniscus, lipid layer images; classification of DED severity	Detection of meibomian gland atrophy and abnormal morphology; DED severity classified in <1 s per image	[43]
AI-assisted patient monitoring	Assessment of therapy effectiveness between visits, early detection of symptom worsening	Mobile apps and high-resolution image analysis allow real-time therapy adjustments; average TBUT improvement of 2.1 s and 20% reduction in OSDI over 3 months	[46]

**Table 2:** Pharmacological treatments in DED. * Evidence level simplified for narrative review purposes (high = multiple RCTs; moderate = small RCTs/cohort; conflicting = inconsistent RCT data). GI—gastrointestinal discomfort.

Therapy	Mechanism of Action	Main Indication	Level of Clinical Evidence *	Major Adverse Effects/Limitations	Stage of Treatment
Artificial tears—demulcents	Hydration, tear film stabilization, dilution of inflammatory mediators	Mild DED	High (widely studied, standard of care)	Temporary relief, requires frequent dosing	First-line
Artificial tears—lipid-based/emollients	Restores lipid layer, reduces evaporation	Evaporative DED, MGD	Moderate–high	Blurred vision (transient), limited anti-inflammatory effect	First-line
Autologous serum	Provides growth factors, immunoglobulins, albumin, lactoferrin	Severe or refractory DED	Moderate (small RCTs, cohort studies)	Preparation complexity, contamination risk, short shelf life	Second-line (severe)
Topical corticosteroids	Broad anti-inflammatory effect (cytokine suppression)	Moderate–severe inflammatory DED (short-term use)	High (short-term efficacy proven)	Increased IOP, cataract, infection risk (long-term use)	Second-line
Cyclosporine A	Inhibits T-cell activation and cytokine release	Chronic inflammatory DED	High (multiple RCTs)	Burning sensation, delayed onset (2–3 months), cost	Second-line
Lifitegrast	Blocks LFA-1/ICAM-1 interaction → reduces T-cell activation	Inflammatory DED	High (phase III RCTs)	Dysgeusia, irritation, modest clinical effect size	Second-line
Tetracyclines (e.g., doxycycline)	Anti-inflammatory, MMP inhibition, improves meibomian gland function	MGD-associated DED	Moderate	GI discomfort, photosensitivity	Second-line (off-label)
Macrolides (e.g., azithromycin)	Anti-inflammatory + improves lipid secretion	MGD-associated DED	Moderate	Local irritation, limited long-term data	Second-line (off-label)
Omega-3 supplementation	Modulates inflammatory lipid mediators	Adjunct therapy (MGD, inflammatory DED)	Conflicting (large RCTs show mixed results)	Uncertain benefit, dosage variability	Adjunctive

**Table 3:** Key clinical studies evaluating pharmacological treatments for DED.

Therapy	Study	Study Design	Sample Size	Main Findings	Clinical Relevance
Cyclosporine A	Sall et al., 2000 [67]	Randomized controlled trial	877 patients	Improved Schirmer test scores and reduced corneal staining	Established CsA as a standard anti-inflammatory treatment
Lifitegrast	OPUS-3 trial [68]	Randomized, double-blind, placebo-controlled	711 patients	Significant improvement in eye dryness score	Effective symptomatic therapy for moderate-to-severe DED
Omega-3 supplements	DREAM study [69]	Multicenter randomized trial	535 patients	No significant difference vs. placebo	Limited evidence for routine supplementation
Doxycycline	Messmer et al., 2015 [55]	Prospective clinical studies	~50–100 patients	Improved meibomian gland function and tear stability	Often used off-label for MGD-related DED

**Table 4:** Emerging and advanced therapies in DED.

Therapy	Development Stage	Study Design/Evidence Level	Proposed Benefit	Clinical Relevance	Key Limitations
Intense pulsed light	Clinically used (off-label)	Small randomized trials, prospective studies	Improves meibomian gland function, reduces inflammation	Promising therapy for MGD-related DED	Cost, protocol variability, limited use in darker skin types
Smart contact lenses	Experimental/early clinical research	Preclinical studies	Continuous biomarker monitoring, controlled drug release	Potential diagnostic and therapeutic platform	Sensor accuracy, oxygen permeability, comfort, power supply issues
Nanoemulsions/microemulsions	Approved (e.g., Clacier) + ongoing development	Preclinical studies and drug-specific clinical trials	Improves bioavailability and corneal residence time	Enhances delivery of lipophilic drugs (e.g., cyclosporine)	Stability issues, droplet aggregation
Liposomes	Clinically available (e.g., Lacrisek)	Small clinical studies, observational studies	Enhanced drug penetration, tear film stabilization	Potential alternative to conventional artificial tears	Limited large RCT data
Nanomicelles	FDA-approved (e.g., Cequa)	Randomized clinical trials for specific drugs	Improves the solubility of lipophilic drugs	Effective delivery system for cyclosporine formulations	Cost, limited comparative trials
Dendrimers	Preclinical/early research	Preclinical animal studies	Targeted drug delivery, anti-inflammatory potential	Potential carrier for ocular drug delivery	Lack of RCTs
Nanowafers	Early clinical phase	Small clinical studies	Sustained drug release, improved adherence	May reduce dosing frequency compared with eye drops	Limited long-term safety data
Salivary gland transplantation	Specialized surgical intervention	Case series, observational studies	Alternative tear source in severe DED	Option for patients with refractory disease	Surgical risk, graft complications
Mesenchymal stem cell therapy	Experimental	Preclinical, early clinical trials	Immunomodulation, lacrimal gland regeneration	Potential regenerative therapy	Inconsistent outcomes, regulatory challenges

**Table 5:** Summary of the most relevant studies evaluating interventional therapies for DED.

Therapy	Study Type	Sample Size	Main Outcomes	Clinical Significance
Punctal plugs	Prospective clinical studies	30–200 patients	Increased tear retention and symptom relief	Common adjunctive therapy
Amniotic membrane (e.g., PROKERA)	Observational studies	50–100 patients	Improved epithelial healing and reduced corneal staining	Useful in moderate–severe DED
Intense pulsed light (IPL)	Small randomized trials	40–90 patients	Improved meibomian gland secretion and TBUT	Promising for MGD-related DED
Tarsorrhaphy	Case series	Small cohorts	Improved ocular surface protection	Reserved for severe cases

## Data Availability

No new data were created or analyzed in this study.

## Abbreviations

The following abbreviations are used in this manuscript:

AI	Artificial intelligence
AM	Amniotic membrane
CNNs	Convolutional neural networks
CsA	Cyclosporine A
DL	Deep learning
DDS	Drug delivery systems
DED	Dry eye disease
FDA	Food and Drug Administration
IOP	Intraocular pressure
IPL	Intense pulsed light
LFA-1	Lymphocyte function-associated antigen-1
LIPCOF scale	Lid-parallel conjunctival folds scale
MHC	Major histocompatibility complex
MGD	Meibomian gland dysfunction
ML	Machine learning
MMPs	Matrix metalloproteinases
MSC	Mesenchymal stem cell
NFC	Near-field communication
NIBUT	Non-invasive tear break-up time
OSDI	Ocular Surface Disease Index
PAMAM-based	Polyamidoamine-based
ROS	Reactive oxygen species
RFID	Radio-frequency identification
SCLs	Smart contact lenses
TBUT	Tear break-up time
TFOS DEWS II	Tear Film and Ocular Surface Society Dry Eye Workshop II
TFOS DEWS III	Tear Film and Ocular Surface Society Dry Eye Workshop III

## References

[1] Britten-Jones A.C., Wang M.T.M., Samuels I., Jennings C., Stapleton F., Craig J.P. (2024). Epidemiology and Risk Factors of Dry Eye Disease: Considerations for Clinical Management. Medicina.

[2] Farrand K.F., Fridman M., Stillman I.Ö., Schaumberg D.A. (2017). Prevalence of Diagnosed Dry Eye Disease in the United States Among Adults Aged 18 Years and Older. Am. J. Ophthalmol..

[3] Craig J.P., Nichols K.K., Akpek E.K., Caffery B., Dua H.S., Joo C.K., Liu Z., Nelson J.D., Nichols J.J., Tsubota K. (2017). TFOS DEWS II Definition and Classification Report. Ocul. Surf..

[4] Pflugfelder S.C., de Paiva C.S. (2017). The Pathophysiology of Dry Eye Disease: What We Know and Future Directions for Research. Ophthalmology.

[5] Perez V.L., Chen W., Craig J.P., Dogru M., Jones L., Stapleton F., Wolffsohn J.S., Sullivan D.A. (2026). TFOS DEWS III: Executive Summary. Am. J. Ophthalmol..

[6] Bron A.J., de Paiva C.S., Chauhan S.K., Bonini S., Gabison E.E., Jain S., Knop E., Markoulli M., Ogawa Y., Perez V. (2017). TFOS DEWS II pathophysiology report. Ocul. Surf..

[7] Lyngstadaas A.V., Olsen M.V., Bair J.A., Hodges R.R., Utheim T.P., Serhan C.N., Dartt D.A. (2021). Pro-Resolving Mediator Annexin A1 Regulates Intracellular Ca^2+^ and Mucin Secretion in Cultured Goblet Cells Suggesting a New Use in Inflammatory Conjunctival Diseases. Front. Immunol..

[8] Yao Y., Zhang Y. (2017). The lacrimal gland: Development, wound repair and regeneration. Biotechnol. Lett..

[9] Egger S.F., Huber-Spitzy V., Böhler K., Scholda C. (1995). Isotretinoinapplikation zur Behandlung der Akne vulgaris. Prospektive Studie über Art und Ausmass der okulären Komplikationen [Isotretinoin administration in treatment of acne vulgaris. A prospective study of the kind and extent of ocular complications]. Ophthalmologe.

[10] Ou S., Zhang L., Wu Y., Yang D., Jiang N., Mao T., Zheng X., Gu H., Zhang L. (2025). Alterations in the composition of meibomian gland secretions in patients with meibomian gland dysfunction based on Raman spectroscopy. Front. Med..

[11] Karpecki P.M., Nichols K.K., Sheppard J.D. (2023). Addressing excessive evaporation: An unmet need in dry eye disease. Am. J. Manag. Care.

[12] Sabeti S., Kheirkhah A., Yin J., Dana R. (2020). Management of meibomian gland dysfunction: A review. Surv. Ophthalmol..

[13] Mansoor H., Tan H.C., Lin M.T., Mehta J.S., Liu Y.C. (2020). Diabetic Corneal Neuropathy. J. Clin. Med..

[14] Amano Y., Sugimoto Y., Sugita M. (2012). Ocular disorders due to eyelash extensions. Cornea.

[15] Choi M.G., Yeo J.H., Kang J.W., Chun Y.S., Lee J.K., Kim J.C. (2019). Effects of botulinum toxin type A on the treatment of dry eye disease and tear cytokines. Graefes Arch. Clin. Exp. Ophthalmol..

[16] Diel R.J., Kroeger Z.A., Levitt R.C., Sarantopoulos C., Sered H., Martinez-Barrizonte J., Galor A. (2018). Botulinum Toxin A for the Treatment of Photophobia and Dry Eye. Ophthalmology.

[17] Tawfik A., Pistilli M., Maguire M.G., Chen Y., Yu Y., Greiner J.V., Asbell P.A., Ying G.S., Dry Eye Assessment and Management (DREAM) Study Research Group (2024). Association of Dry Eye Symptoms and Signs in Patients with Dry Eye Disease. Ophthalmic Epidemiol..

[18] Vehof J., Sillevis Smitt-Kamminga N., Nibourg S.A., Hammond C.J. (2017). Predictors of Discordance between Symptoms and Signs in Dry Eye Disease. Ophthalmology.

[19] Kawahara A. (2023). Treatment of Dry Eye Disease (DED) in Asia: Strategies for Short Tear Film Breakup Time-Type DED. Pharmaceutics.

[20] Szczotka-Flynn L.B., Maguire M.G., Ying G.S., Lin M.C., Bunya V.Y., Dana R., Asbell P.A., Dry Eye Assessment and Management (DREAM) Study Research Group (2019). Impact of Dry Eye on Visual Acuity and Contrast Sensitivity: Dry Eye Assessment and Management Study. Optom. Vis. Sci..

[21] Kandel H., Stapleton F.A.O., Downie L.E., Chidi-Egboka N.C., MIngo-Botin D., Arnalich-Montiel F., Rauz S., Recchioni A., Sitaula S., Markoulli M. (2025). The impact of dry eye disease on patient-reported quality of life: A Save Sight Dry Eye Registry study. Ocul. Surf..

[22] Stapleton F., Velez F.G., Lau C., Wolffsohn J.S. (2024). Dry eye disease in the young: A narrative review. Ocul. Surf..

[23] Ghach W., Bakkar M.M., Aridi M., Alebrahim M.A. (2025). Symptomatic dry eye disease (DED) in cohort of contact lens wearers in Jordan. PLoS ONE.

[24] Al-Mohtaseb Z., Schachter S., Shen Lee B., Garlich J., Trattler W. (2021). The Relationship Between Dry Eye Disease and Digital Screen Use. Clin. Ophthalmol..

[25] Zou Y., Li D., Gianni V., Congdon N., Piyasena P., Prakalapakorn S.G., Zhang R., Zhao Z., Chan V.F., Yu M. (2025). Prevalence of dry eye disease among children: A systematic review and meta-analysis. BMJ Open Ophthalmol..

[26] Basilious A., Xu C.Y., Malvankar-Mehta M.S. (2022). Dry eye disease and psychiatric disorders: A systematic review and meta-analysis. Eur. J. Ophthalmol..

[27] Villani E., Barabino S., Giannaccare G., Di Zazzo A., Aragona P., Rolando M. (2025). From Symptoms to Satisfaction: Optimizing Patient-Centered Care in Dry Eye Disease. J. Clin. Med..

[28] Borrelli M., Frings A., Geerling G., Finis D. (2021). Gender-Specific Differences in Signs and Symptoms of Dry Eye Disease. Curr. Eye Res..

[29] Wu K., Yu Y., Shi J., Chen H., Xie C., Tang Y., Yao X. (2025). Relationship between myopia and diagnosis rates of dry eye disease and related indicators: A systematic review and meta-analysis. Front. Med..

[30] Guo M., Diaz G.M., Yu Y., Patel C.A., Farrar J.T., Asbell P.A., Ying G.S., Dry Eye Assessment and Management Study Research Group (2024). Association between systemic medication use and severity of dry eye signs and symptoms in the DRy eye assessment and management (DREAM) study. Ocul. Surf..

[31] Shimazaki J. (2018). Definition and Diagnostic Criteria of Dry Eye Disease: Historical Overview and Future Directions. Investig. Ophthalmol. Vis. Sci..

[32] Tsubota K., Pflugfelder S.C., Liu Z., Baudouin C., Kim H.M., Messmer E.M., Kruse F., Liang L., Carreno-Galeano J.T., Rolando M. (2020). Defining Dry Eye from a Clinical Perspective. Int. J. Mol. Sci..

[33] Fasciani R., Troisi S., Troisi M., Odorici S., Versura P. (2025). A Narrative Review on the Diagnosis of Dry Eye Disease: Insights from the Italian Dry Eye Consensus (IDEC) Group. Ophthalmol. Ther..

[34] Chester T., Garg S.S., Johnston J., Ayers B., Gupta P. (2023). How Can We Best Diagnose Severity Levels of Dry Eye Disease: Current Perspectives. Clin. Ophthalmol..

[35] Elhusseiny A.M., Khalil A.A., El Sheikh R.H., Bakr M.A., Eissa M.G., El Sayed Y.M. (2019). New approaches for diagnosis of dry eye disease. Int. J. Ophthalmol..

[36] Wu Y., Wang C., Wang X., Mou Y., Yuan K., Huang X., Jin X. (2022). Advances in Dry Eye Disease Examination Techniques. Front. Med..

[37] Bron A.J., Abelson M.B., Ousler G., Pearce E., Tomlinson A., Yokoi N., Smith J.A., Begley C., Caffery B., Nichols K. (2007). Methodologies to diagnose and monitor dry eye disease: Report of the Diagnostic Methodology Subcommittee of the International Dry Eye WorkShop (2007). Ocul. Surf..

[38] Bandlitz S., Purslow C., Murphy P.J., Pult H. (2019). Lid-parallel conjunctival fold (LIPCOF) morphology imaged by optical coherence tomography and its relationship to LIPCOF grade. Cont. Lens Anterior Eye.

[39] Park J., Choi Y., Han G., Shin E., Han J., Chung T.Y., Lim D.H. (2021). Evaluation of tear osmolarity measured by I-Pen osmolarity system in patients with dry eye. Sci. Rep..

[40] Kojima T., Dogru M., Kawashima M., Nakamura S., Tsubota K. (2020). Advances in the diagnosis and treatment of dry eye. Prog. Retin. Eye Res..

[41] Han S.B., Liu Y.C., Mohamed-Noriega K., Tong L., Mehta J.S. (2020). Objective Imaging Diagnostics for Dry Eye Disease. J. Ophthalmol..

[42] Barrientos R.T., Godín F., Rocha-De-Lossada C., Soifer M., Sánchez-González J.M., Moreno-Toral E., González A.L., Zein M., Larco PJr Mercado C., Piedrahita M.A. (2022). Ophthalmological Approach for the Diagnosis of Dry Eye Disease in Patients with Sjögren’s Syndrome. Life.

[43] Storås A.M., Strümke I., Riegler M.A., Grauslund J., Hammer H.L., Yazidi A., Halvorsen P., Gundersen K.G., Utheim T.P., Jackson C.J. (2022). Artificial intelligence in dry eye disease. Ocul. Surf..

[44] Shimizu E., Ishikawa T., Tanji M., Agata N., Nakayama S., Nakahara Y., Yokoiwa R., Sato S., Hanyuda A., Ogawa Y. (2023). Artificial intelligence to estimate the tear film breakup time and diagnose dry eye disease. Sci. Rep..

[45] Yang H.K., Che S.A., Hyon J.Y., Han S.B. (2022). Integration of Artificial Intelligence into the Approach for Diagnosis and Monitoring of Dry Eye Disease. Diagnostics.

[46] Lu M., Yang K., Deng X., Fan T., Zhang H., Yang W., Xing Y. (2025). Artificial Intelligence in Clinical Diagnosis and Treatment of Dry Eye: Workflows, Effectiveness, and Evaluation. J. Curr. Ophthalmol..

[47] Jones L., Downie L.E., Korb D., Benitez-Del-Castillo J.M., Dana R., Deng S.X., Dong P.N., Geerling G., Hida R.Y., Liu Y. (2017). TFOS DEWS II Management and Therapy Report. Ocul. Surf..

[48] Harti M.E., Andaloussi S.J., Ouchetto O. (2025). A Comprehensive Review Comparing Artificial Intelligence and Clinical Diagnostic Approaches for Dry Eye Disease. Diagnostics.

[49] Costin H.-N., Fira M., Goraș L. (2025). Artificial Intelligence in Ophthalmology: Advantages and Limits. Appl. Sci..

[50] Nair P.P., Keskar M., Borghare P.T., Methwani D.A., Nasre Y., Chaudhary M. (2024). Artificial Intelligence in Dry Eye Disease: A Narrative Review. Cureus.

[51] Shen Lee B., Kabat A.G., Bacharach J., Karpecki P., Luchs J. (2020). Managing Dry Eye Disease and Facilitating Realistic Patient Expectations: A Review and Appraisal of Current Therapies. Clin. Ophthalmol..

[52] Aragona P., Giannaccare G., Mencucci R., Rubino P., Cantera E., Rolando M. (2021). Modern approach to the treatment of dry eye, a complex multifactorial disease: A P.I.C.A.S.S.O. board review. Br. J. Ophthalmol..

[53] Pucker A.D., Ng S.M., Nichols J.J. (2016). Over the counter (OTC) artificial tear drops for dry eye syndrome. Cochrane Database Syst. Rev..

[54] Kathuria A., Shamloo K., Jhanji V., Sharma A. (2021). Categorization of Marketed Artificial Tear Formulations Based on Their Ingredients: A Rational Approach for Their Use. J. Clin. Med..

[55] Messmer E.M. (2015). The pathophysiology, diagnosis, and treatment of dry eye disease. Dtsch. Arztebl. Int..

[56] Milner M.S., Beckman K.A., Luchs J.I., Allen Q.B., Awdeh R.M., Berdahl J., Boland T.S., Buznego C., Gira J.P., Goldberg D.F. (2017). Dysfunctional tear syndrome: Dry eye disease and associated tear film disorders-new strategies for diagnosis and treatment. Curr. Opin. Ophthalmol..

[57] Bian X., Ma J., Liu Y., Feng Y., Liu Z., Zhang B., Huang B. (2025). Cyclosporine a in the treatment of dry eye disease: A narrative review. Front. Ophthalmol..

[58] De Paiva C.S., Corrales R.M., Villarreal A.L., Farley W., Li D.Q., Stern M.E., Pflugfelder S.C. (2006). Apical corneal barrier disruption in experimental murine dry eye is abrogated by methylprednisolone and doxycycline. Investig. Ophthalmol. Vis. Sci..

[59] Kagkelaris K.A., Makri O.E., Georgakopoulos C.D., Panayiotakopoulos G.D. (2018). An eye for azithromycin: Review of the literature. Ther. Adv. Ophthalmol..

[60] Ben Ephraim Noyman D., Chan C.C., Mimouni M., Safir M. (2024). Systemic antibiotic treatment for meibomian gland dysfunction-A systematic review and meta-analysis. Acta Ophthalmol..

[61] Li J.X., Tsai Y.Y., Lai C.T., Li Y.L., Wu Y.H., Chiang C.C. (2022). Lifitegrast Ophthalmic Solution 5% Is a Safe and Efficient Eyedrop for Dry Eye Disease: A Systematic Review and Meta-Analysis. J. Clin. Med..

[62] Hovanesian J.A., Nichols K.K., Jackson M., Katz J., Chan A., Glassberg M.B., Sloesen B., Korves C., Nguyen C., Syntosi A. (2021). Real-World Experience with Lifitegrast Ophthalmic Solution (Xiidra^®^) in the US and Canada: Retrospective Study of Patient Characteristics, Treatment Patterns, and Clinical Effectiveness in 600 Patients with Dry Eye Disease. Clin. Ophthalmol..

[63] Abidi A., Shukla P., Ahmad A. (2016). Lifitegrast: A novel drug for treatment of dry eye disease. J. Pharmacol. Pharmacother..

[64] Garrigue J.S., Amrane M., Faure M.O., Holopainen J.M., Tong L. (2017). Relevance of Lipid-Based Products in the Management of Dry Eye Disease. J. Ocul. Pharmacol. Ther..

[65] Liu A., Ji J. (2014). Omega-3 essential fatty acids therapy for dry eye syndrome: A meta-analysis of randomized controlled studies. Med. Sci. Monit..

[66] Christen W.G., Cook N.R., Manson J.E., Buring J.E., Lee I.M., Bubes V., Friedenberg G., Dushkes R., Smith D., Schaumberg D.A. (2022). Efficacy of Marine ω-3 Fatty Acid Supplementation vs Placebo in Reducing Incidence of Dry Eye Disease in Healthy US Adults: A Randomized Clinical Trial. JAMA Ophthalmol..

[67] Sall K., Stevenson O.D., Mundorf T.K., Reis B.L. (2000). Two multicenter, randomized studies of the efficacy and safety of cyclosporine ophthalmic emulsion in moderate to severe dry eye disease. CsA Phase 3 Study Group. Ophthalmology.

[68] Holland E.J., Luchs J., Karpecki P.M., Nichols K.K., Jackson M.A., Sall K., Tauber J., Roy M., Raychaudhuri A., Shojaei A. (2017). Lifitegrast for the Treatment of Dry Eye Disease: Results of a Phase III, Randomized, Double-Masked, Placebo-Controlled Trial (OPUS-3). Ophthalmology.

[69] Asbell P.A., Maguire M.G., Peskin E., Bunya V.Y., Kuklinski E.J., Dry Eye Assessment and Management (DREAM©) Study Research Group (2018). Dry Eye Assessment and Management (DREAM©) Study: Study design and baseline characteristics. Contemp. Clin. Trials..

[70] Jehangir N., Bever G., Mahmood S.M., Moshirfar M. (2016). Comprehensive Review of the Literature on Existing Punctal Plugs for the Management of Dry Eye Disease. J. Ophthalmol..

[71] Mead O.G., Tighe S., Tseng S.C.G. (2020). Amniotic membrane transplantation for managing dry eye and neurotrophic keratitis. Taiwan J. Ophthalmol..

[72] Rajak S., Rajak J., Selva D. (2015). Performing a tarsorrhaphy. Community Eye Health.

[73] O’Neil E.C., Henderson M., Massaro-Giordano M., Bunya V.Y. (2019). Advances in dry eye disease treatment. Curr. Opin. Ophthalmol..

[74] Qin G., Chen J., Li L., Zhang Q., Xu L., Yu S., He W., He X., Pazo E.E. (2023). Efficacy of intense pulsed light therapy on signs and symptoms of dry eye disease: A meta-analysis and systematic review. Indian J. Ophthalmol..

[75] Kim J., Kim M., Lee M.S., Kim K., Ji S., Kim Y.T., Park J., Na K., Bae K.H., Kim H.K. (2017). Wearable smart sensor systems integrated on soft contact lenses for wireless ocular diagnostics. Nat. Commun..

[76] Yin R., Xu Z., Mei M., Chen Z., Wang K., Liu Y., Tang T., Priydarshi M.K., Meng X., Zhao S. (2018). Soft transparent graphene contact lens electrodes for conformal full-cornea recording of electroretinogram. Nat. Commun..

[77] Badugu R., Lakowicz J.R., Geddes C.D. (2003). A Glucose Sensing Contact Lens: A Non-Invasive Technique for Continuous Physiological Glucose Monitoring. J. Fluoresc..

[78] Park W., Seo H., Kim J., Hong Y.M., Song H., Joo B.J., Kim S., Kim E., Yae C.G., Kim J. (2024). In-depth correlation analysis between tear glucose and blood glucose using a wireless smart contact lens. Nat. Commun..

[79] Kim S.K., Lee G.H., Jeon C., Han H.H., Kim S.J., Mok J.W., Joo C.K., Shin S., Sim J.Y., Myung D. (2022). Bimetallic Nanocatalysts Immobilized in Nanoporous Hydrogels for Long-Term Robust Continuous Glucose Monitoring of Smart Contact Lens. Adv. Mater..

[80] Yang C., Wu Q., Liu J., Mo J., Li X., Yang C., Liu Z., Yang J., Jiang L., Chen W. (2022). Intelligent wireless theranostic contact lens for electrical sensing and regulation of intraocular pressure. Nat. Commun..

[81] Kim J., Cha E., Park J.U. (2020). Recent Advances in Smart Contact Lenses. Adv. Mater. Technol..

[82] Kim T.Y., Shin S., Choi H., Jeong S.H., Myung D., Hahn S.K. (2021). Smart Contact Lenses with a Transparent Silver Nanowire Strain Sensor for Continuous Intraocular Pressure Monitoring. ACS Appl. Bio Mater..

[83] Xu J., Cui T., Hirtz T., Qiao Y., Li X., Zhong F., Han X., Yang Y., Zhang S., Ren T.L. (2020). Highly Transparent and Sensitive Graphene Sensors for Continuous and Non-invasive Intraocular Pressure Monitoring. ACS Appl. Mater. Interfaces.

[84] Zhang H., Zhu X., Tai Y., Zhou J., Li H., Li Z., Wang R., Zhang J., Zhang Y., Ge W. (2023). Recent advances in nanofiber-based flexible transparent electrodes. Int. J. Extrem. Manuf..

[85] Sun R., Zhang J., Chen X., Deng Y., Gou J., Yin T., He H., Tang X., Ni X., Yang L. (2024). An adaptive drug-releasing contact lens for personalized treatment of ocular infections and injuries. J. Control. Release.

[86] Keum D.H., Kim S.K., Koo J., Lee G.H., Jeon C., Mok J.W., Mun B.H., Lee K.J., Kamrani E., Joo C.K. (2020). Wireless smart contact lens for diabetic diagnosis and therapy. Sci. Adv..

[87] Dunbar G.E., Shen B.Y., Aref A.A. (2017). The Sensimed Triggerfish contact lens sensor: Efficacy, safety, and patient perspectives. Clin. Ophthalmol..

[88] Seo H., Chung W.G., Kwon Y.W., Kim S., Hong Y.M., Park W., Kim E., Lee J., Lee S., Kim M. (2023). Smart Contact Lenses as Wearable Ophthalmic Devices for Disease Monitoring and Health Management. Chem. Rev..

[89] Chiou J.C., Hsu S.H., Liao Y.T., Huang Y.C., Yeh G.T., Kuei C.K., Dai K.S. (2016). Toward a Wirelessly Powered On-Lens Intraocular Pressure Monitoring System. IEEE J. Biomed. Health Inf..

[90] (2019). IEEE Standard for Safety Levels with Respect to Human Exposure to Electric, Magnetic, and Electromagnetic Fields, 0 Hz to 300 GHz.

[91] Park J., Ahn D.B., Kim J., Cha E., Bae B.S., Lee S.Y., Park J.U. (2019). Printing of wirelessly rechargeable solid-state supercapacitors for soft, smart contact lenses with continuous operations. Sci. Adv..

[92] Lee H.S., Kim S., Kim K.B., Choi J.W. (2018). Scalable fabrication of flexible thin-film batteries for smart lens applications. Nano Energy.

[93] Yun J., Zeng Y., Kim M., Gao C., Kim Y., Lu L., Kim T.T., Zhao W., Bae T.H., Lee S.W. (2021). Tear-Based Aqueous Batteries for Smart Contact Lenses Enabled by Prussian Blue Analogue Nanocomposites. Nano Lett..

[94] Park S., Heo S.W., Lee W., Inoue D., Jiang Z., Yu K., Jinno H., Hashizume D., Sekino M., Yokota T. (2018). Self-powered ultra-flexible electronics via nano-grating-patterned organic photovoltaics. Nature.

[95] Lin B., Wang M., Zhao C., Wang S., Chen K., Li X., Long Z., Zhao C., Song X., Yan S. (2022). Flexible organic integrated electronics for self-powered multiplexed ocular monitoring. npj Flex. Electron..

[96] Pu X., Zhang C., Wang Z.L. (2022). Triboelectric nanogenerators as wearable power sources and self-powered sensors. Natl. Sci. Rev..

[97] Pourshaban E., Karkhanis M.U., Deshpande A., Banerjee A., Hasan M.R., Nikeghbal A., Ghosh C., Kim H., Mastrangelo C.H. (2024). Power Scavenging Microsystem for Smart Contact Lenses. Small.

[98] Phan C.M., Subbaraman L., Jones L.W. (2016). The Use of Contact Lenses as Biosensors. Optom. Vis. Sci..

[99] Nagai N., Otake H. (2022). Novel drug delivery systems for the management of dry eye. Adv. Drug Deliv. Rev..

[100] Coursey T.G., Wassel R.A., Quiambao A.B., Farjo R.A. (2018). Once-Daily Cyclosporine-A-MiDROPS for Treatment of Dry Eye Disease. Transl. Vis. Sci. Technol..

[101] Daull P., Lallemand F., Garrigue J.S. (2014). Benefits of cetalkonium chloride cationic oil-in-water nanoemulsions for topical ophthalmic drug delivery. J. Pharm. Pharmacol..

[102] Kaur I.P., Garg A., Singla A.K., Aggarwal D. (2004). Vesicular systems in ocular drug delivery: An overview. Int. J. Pharm..

[103] McCann L.C., Tomlinson A., Pearce E.I., Papa V. (2012). Effectiveness of artificial tears in the management of evaporative dry eye. Cornea.

[104] Linares-Alba M.A., Gómez-Guajardo M.B., Fonzar J.F., Brooks D.E., García-Sánchez G.A., Bernad-Bernad M.J. (2016). Preformulation Studies of a Liposomal Formulation Containing Sirolimus for the Treatment of Dry Eye Disease. J. Ocul. Pharmacol. Ther..

[105] Richichi B., Baldoneschi V., Burgalassi S., Fragai M., Vullo D., Akdemir A., Dragoni E., Louka A., Mamusa M., Monti D. (2016). A Divalent PAMAM-Based Matrix Metalloproteinase/Carbonic Anhydrase Inhibitor for the Treatment of Dry Eye Syndrome. Chemistry.

[106] Tawfik S.M., Azizov S., Elmasry M.R., Sharipov M., Lee Y.I. (2020). Recent Advances in Nanomicelles Delivery Systems. Nanomaterials.

[107] Shen Y., Yu Y., Chaurasiya B., Li X., Xu Y., Webster T.J., Tu J., Sun R. (2018). Stability, safety, and transcorneal mechanistic studies of ophthalmic lyophilized cyclosporine-loaded polymeric micelles. Int. J. Nanomed..

[108] Mandal A., Gote V., Pal D., Ogundele A., Mitra A.K. (2019). Ocular Pharmacokinetics of a Topical Ophthalmic Nanomicellar Solution of Cyclosporine (Cequa^®^) for Dry Eye Disease. Pharm. Res..

[109] Mehra N.K., Cai D., Kuo L., Hein T., Palakurthi S. (2016). Safety and toxicity of nanomaterials for ocular drug delivery applications. Nanotoxicology.

[110] Coursey T.G., Henriksson J.T., Marcano D.C., Shin C.S., Isenhart L.C., Ahmed F., De Paiva C.S., Pflugfelder S.C., Acharya G. (2015). Dexamethasone nanowafer as an effective therapy for dry eye disease. J. Control. Release.

[111] Yuan X., Marcano D.C., Shin C.S., Hua X., Isenhart L.C., Pflugfelder S.C., Acharya G. (2015). Ocular drug delivery nanowafer with enhanced therapeutic efficacy. ACS Nano.

[112] Joshi V.P., Singh S., Thacker M., Pati F., Vemuganti G.K., Basu S., Singh V. (2023). Newer approaches to dry eye therapy: Nanotechnology, regenerative medicine, and tissue engineering. Indian J. Ophthalmol..

[113] Walker M.K., Bergmanson J.P., Miller W.L., Marsack J.D., Johnson L.A. (2016). Complications and fitting challenges associated with scleral contact lenses: A review. Cont. Lens Anterior Eye.

[114] Chen J., Bai T., Su J., Cong X., Lv L., Tong L., Yu H., Feng Y., Yu G. (2024). Salivary Gland Transplantation as a Promising Approach for Tear Film Restoration in Severe Dry Eye Disease. J. Clin. Med..

[115] Soleimani M., Masoumi A., Momenaei B., Cheraqpour K., Koganti R., Chang A.Y., Ghassemi M., Djalilian A.R. (2023). Applications of mesenchymal stem cells in ocular surface diseases: Sources and routes of delivery. Expert. Opin. Biol. Ther..

[116] Jiang Y., Lin S., Gao Y. (2022). Mesenchymal Stromal Cell-Based Therapy for Dry Eye: Current Status and Future Perspectives. Cell Transpl..

[117] Sanabria-de la Torre R., Quiñones-Vico M.I., Fernández-González A., Sánchez-Díaz M., Montero-Vílchez T., Sierra-Sánchez Á., Arias-Santiago S. (2021). Alloreactive Immune Response Associated to Human Mesenchymal Stromal Cells Treatment: A Systematic Review. J. Clin. Med..

[118] Galipeau J., Sensébé L. (2018). Mesenchymal Stromal Cells: Clinical Challenges and Therapeutic Opportunities. Cell Stem Cell.

[119] Awad H., Abas M., Elgharably H., Tripathi R., Theofilos T., Bhandary S., Sai-Sudhakar C., Sen C.K., Roy S. (2012). Endogenous opioids in wound-site neutrophils of sternotomy patients. PLoS ONE.

[120] Mohamed H.B., Abd El-Hamid B.N., Fathalla D., Fouad E.A. (2022). Current trends in pharmaceutical treatment of dry eye disease: A review. Eur. J. Pharm. Sci..

[121] Deveney T., Asbell P.A. (2018). Patient and physician perspectives on the use of cyclosporine ophthalmic emulsion 0.05% for the management of chronic dry eye. Clin. Ophthalmol..

[122] Lallemand F., Schmitt M., Bourges J.L., Gurny R., Benita S., Garrigue J.S. (2017). Cyclosporine A delivery to the eye: A comprehensive review of academic and industrial efforts. Eur. J. Pharm. Biopharm..

